# Changes in Protein Metabolism and Early Development of Sarcopenia in Mice With Cholestatic Liver Disease

**DOI:** 10.1002/jcsm.13737

**Published:** 2025-02-19

**Authors:** Ottavia Agrifoglio, Solvig Görs, Miriama Sciascia, Zeyang Li, Elke Albrecht, Sophie Achilles, Meike Statz, Manuela Bastian, Tobias Lindner, Karen Friederike Gauß, Sarah Rohde, Karen Rischmüller, Peggy Berlin, Georg Lamprecht, Robert Jaster, Cornelia C. Metges, Luise Ehlers

**Affiliations:** ^1^ Nutrition and Metabolism Research Institute for Farm Animal Biology (FBN) Dummerstorf Germany; ^2^ Department of Medicine II, Division of Gastroenterology and Endocrinology Rostock University Medical Center Rostock Germany; ^3^ Institute of Clinical Chemistry and Laboratory Medicine Rostock University Medical Center Rostock Germany; ^4^ Core Facility Multimodal Small Animal Imaging Rostock University Medical Center Rostock Germany; ^5^ Institute of Clinical Chemistry and Laboratory Medicine University Medicine Greifswald Greifswald Germany; ^6^ University Institute of Clinical Chemistry and Laboratory Medicine University Oldenburg Oldenburg Germany

**Keywords:** cholestatic liver disease, malnutrition, metabolism, mouse model, sarcopenia

## Abstract

**Background:**

Sarcopenia is a frequent complication of liver cirrhosis. Here, we chose a mouse model of cholestatic liver disease (CLD) to gain mechanistic insights into the development of sarcopenia from the earliest stages of chronic liver injury. Particular attention was paid to protein metabolism, metabolite profiles, and mediators of CLD‐induced muscle wasting.

**Methods:**

Male C57BL/6 J mice underwent bile duct ligation (BDL), sham surgery, or served as untreated controls. The observation phase lasted from the preoperative stage to postoperative day 14. Metabolic cage experiments were performed to determine the nitrogen balance (N‐BAL), nitrogen metabolite profiles, and total energy expenditure (TEE) using doubly labelled water. The fractional protein synthesis rate (FPSR) was assessed using ^2^H_5_‐ring‐phenylalanine. Plasma concentrations of inflammatory markers, metabolites, and enzymes associated with liver damage were investigated. Muscle strength and volume were assessed using a grip strength meter and MRI, respectively. Gene expression was analysed by real‐time PCR.

**Results:**

BDL caused CLD with necroses and inflammation, increased bilirubin (*p* < 0.0001) and conjugated bile acids (*p* < 0.05), and reduced food intake (*p* < 0.0001) and body weight (*p* < 0.0001; each vs. sham). Compared to controls, BDL mice showed lower N‐BAL (*p* < 0.05), reduced TEE (*p* < 0.01), and lower FPSR in the liver (*p* < 0.05) and quadriceps muscle (*p* < 0.001). Arginine was the only plasma amino acid that was diminished after BDL compared to controls and sham treatment (*p* < 0.0001). Reduced muscle strength was observed as early as d3/d4 after BDL (*p* < 0.001; vs. sham), while muscle volume decreased from d6 to d13 (*p* < 0.05). In quadriceps muscle, a lower nuclei‐to‐fibre ratio (*p* < 0.001) and elevated 1‐methyl‐histidine (1‐MH) (*p* < 0.001) were detected, whereas 3‐MH was increased in the urine of BDL mice (*p* < 0.001; each vs. sham). The quadriceps muscle of BDL mice contained higher mRNA levels of atrophy‐associated genes (*Trim63*: *p* < 0.0001, *Fbxo32*: *p* < 0.01) and *Mstn* (*p* < 0.05), but lower levels of genes involved in mitochondrial function (*Cpt‐1b*: *p* < 0.05, *Pgc‐1α*: *p* < 0.01; each vs. sham). In the plasma of BDL mice, elevated protein levels of TNF receptor‐1 (*p* < 0.0001) and HGF‐1 (*p* < 0.05) were observed, while myostatin was diminished (*p* < 0.05; each vs. sham).

**Conclusions:**

Sarcopenia occurs early in CLD and is a multicausal process. Relevant pathophysiologies include reduced protein synthesis, degradation of muscle proteins, arginine deficiency, a systemic pro‐inflammatory and catabolic state, and muscle toxicity of bile acids. Consequently, the treatment of sarcopenia should focus both on eliminating the cause of the cholestasis and on symptomatic measures such as anti‐inflammatory treatment, lowering the bile acid level, and targeted compensation of deficiencies.

## 1 Introduction

The liver is the central regulatory organ of glucose, fatty acid, and amino acid (AA) metabolism. Chronic insults to the liver can result in impaired function due to inflammation, necrosis, fibrosis, hepatocellular dysfunction, and vascular remodelling, eventually culminating in liver cirrhosis (LC) [1]. LC represents the common end‐stage syndrome of different underlying diseases, including cholestatic liver disease (CLD). The most common causes of CLD are primary biliary cholangitis (PBC) and primary sclerosing cholangitis (PSC). Other important pathologies include drug‐induced cholestasis, inflammatory cholestasis, and hereditary cholestasis syndromes [2].

LC accounts for 2–4% of deaths worldwide and, in 2017, caused 1.32 million deaths globally [3]. One of the comorbidities of cirrhosis is poor nutritional status, with the prevalence of malnutrition in cirrhotic patients ranging from 20 to 90% [2]. The aetiology of cirrhotic malnutrition is multifactorial, but the type of malnourishment in LC is primarily a protein‐caloric subtype [4]. This is caused by decreased food intake due to a lack of appetite and gastrointestinal conditions mediated by a pro‐inflammatory state and systemic inflammation affecting appetite and food intake [5]. Up to 70% of LC patients are also affected by a decrease in muscle mass and function [6], which often manifests clinically in parallel with malnutrition as malnutrition‐sarcopenia syndrome [7]. Severe sarcopenia in LC patients is related to increased complications both before and after liver transplantation [8]. The pathogenesis of sarcopenia in LC along the liver‐muscle axis is multifactorial and mechanistically not well understood. Established factors include physical inactivity, insufficient energy intake, altered macronutrient metabolism, and systemic inflammation [7]. It has been shown that decreased levels of testosterone and insulin‐like growth factor‐1 (IGF‐1) contribute to increased myostatin expression and impaired protein synthesis [9]. The imbalance of protein synthesis and proteolysis is also triggered, and maintained, by further mediators of the liver‐muscle axis, such as hyperammonemia, endotoxemia, and insulin resistance [7, 10].

In rodents, bile duct ligation (BDL) causes CLD, liver fibrosis, and cirrhosis. Development of LC in bile duct‐ligated mice and rats is accompanied by progressive sarcopenia [11, 12]. Here, we used the model of BDL in mice to study the development of sarcopenia from the onset of liver injury. To gain mechanistic insights into the early stage of sarcopenia, special attention was paid to changes in protein metabolism and metabolite profiles, as well as the identification of potential mediators of CLD‐induced muscle damage. The results suggest that sarcopenia develops under conditions of a characteristic profile of metabolites and mediators that differs from the pattern of established LC.

## 2 Materials and Methods

### 2.1 Animal Studies

C57BL/6J mice were purchased from Charles River Laboratories (Sulzfeld, Germany) and bred in the local animal facility of the Rostock University Medical Center. The study protocol was approved by the Landesamt für Landwirtschaft, Lebensmittelsicherheit und Fischerei Mecklenburg‐Vorpommern (LALLF/MV; approval no. 7221.3‐1.1‐046/18). All animals were treated following the German Animal Protection Act (TierSchG) and the European Union Directive, 2010/63/EU. The mice had *ad libitum* access to water and rodent diet (V1534‐0; 19% crude protein, 3.3% crude fat, 4.9% crude fibre, 16.3 MJ ME/kg diet; ssniff Spezialdiäten GmbH, Soest, Germany). One week before surgery until the end of the experimental period, 50 g of feed was provided daily as a mixture of 30% dry V1534‐0 feed and 70% water. Mice were single‐housed during the experiments.

At approximately 17 weeks of age, male mice were randomly assigned to the experimental groups BDL, sham surgery, and no surgery (controls). Before surgery, the mice were anaesthetised with isoflurane (5% for induction, 1.5% for maintenance), and carprofen (Pfizer GmbH, Berlin, Germany) at 5 mg/kg body weight (BW) was injected subcutaneously for pain prevention. Analgesia was continued with metamizol (Ratiopharm GmbH, Ulm, Germany) dissolved in drinking water (1.25 mg/mL) until the end of the experiment. BDL and sham surgery were performed as previously described [13].

On days 7–14 after surgery (as specified below), the animals were sacrificed by an overdose of ketamine/xylazine hydrochloride followed by cervical dislocation. The required tissues were collected, and their wet weights were determined. Blood was collected from the retro‐orbital venous sinus in Lithium heparin tubes and centrifuged at 3000 × g for 20 min at 4°C to obtain plasma. All samples were stored under appropriate conditions until they were assayed as detailed below.

### 2.2 Histology

Haematoxylin and eosin (H&E) staining of liver tissue was performed on 4 μm sections of formalin‐fixed, paraffin‐embedded (FFPE) material using standard procedures. For collagen detection, Sirius red staining was performed as previously described [14].

The quadriceps femoris and gastrocnemius muscles ( *M. quadriceps* and *M. gastrocnemius*) were collected, weighed, snap‐frozen in liquid nitrogen, and stored at −80°C. The muscles were sliced at 12 μm thickness using a cryostat microtome (CM3050 S, Leica, Bensheim, Germany), and stained and analysed as described by Zitnan et al. [15] To count nuclei and fibres, the H&E‐stained samples were photographed in six randomly selected areas, using a light microscope with an integrated camera (Nikon Microphot SA, Nikon, Tokyo, Japan) and a 20 x objective. H&E‐stained sections were additionally used to measure the muscle fibre size using an Olympus BX43 microscope (Olympus, Hamburg, Germany) with a DP23 camera and the muscle fibre measurement module of CellSens Dimension version 3.2 (Evident, Hamburg, Germany). At least 200 cells were measured in three randomly selected regions.

To determine the ratio of capillaries to fibres, tissue sections were fixed in a 4% formaldehyde‐Ca^2+^ solution for 5 min, rinsed, and incubated for 90 min at 37°C in a solution with a pH of 9.4 containing 3% Na‐β‐glycerophosphate, 2% sodium 5,5‐diethyl barbiturate, 2% CaCl_2_, and 5% MgSO_4_, and distilled water. Subsequently, the sections were stained with cobalt chloride, rinsed, stained with ammonium sulphide, rinsed, stained with eosin, and left in distilled water for at least 3 h to remove excess dye. The slides were photographed with the same device as described above but using a 10 x objective. Nuclei, fibres, and capillaries were counted using the Cell^F imaging software version 3.4 (OSIS, Münster, Germany) and capillary‐to‐fibre and nuclei‐to‐fibre ratios were calculated.

The presence of intramyocellular lipid droplets was assessed by Oil Red O staining in serial quadriceps muscle sections using standard procedures. The images were taken with an Olympus BX43 microscope equipped with a UC30 camera and CellSens software. The total cross‐sectional area of the muscle was measured using the 1.25 x objective and the interactive polygon function of the CellSense software. Images of all regions with visible lipid droplets from three slices were taken with the 10 x objective. The total lipid area was measured using CellSense software and expressed as the percentage of the total cross‐sectional area.

### 2.3 Muscle Strength

Skeletal muscle strength was determined non‐invasively using a BIO‐GS3 grip strength meter (Bioseb, Vitrolles, France). For this purpose, the device was set up horizontally, and the mice were held by the tail and lowered towards the T‐bar of the device. The mice were allowed to grab the triangular pull rod with the two front paws and were then pulled backward in the horizontal plane. The force exerted on the rod just before the mouse lost its grip was recorded as peak tension. Each animal was measured three times per time point and the mean value was used for further calculations.

### 2.4 Magnetic Resonance Imaging (MRI) of the *M. quadriceps*

MRI measurements of the *M. quadriceps* were performed for all animals at three time points: one day before surgery (d‐1), 6 days after surgery (d6), and 13 days after surgery (d13). The technical details are described in the Supplemental Experimental Procedures section.

### 2.5 Nitrogen Balance (N‐BAL) and Total Energy Expenditure (TEE) Experiments

Before and after BDL or sham surgery, mice were individually placed in metabolic cages (TECNIPLAST GmbH, Hohenpeißenberg, Germany), three times for 24 h each: from d6 to d5 before surgery (d‐6/‐5), from d2 to d3 after surgery (d2/3), and from d9 to d10 after surgery (d9/10). For the determination of N‐BAL and TEE, please refer to the Supplemental Experimental Procedures. Feed and water intake were monitored individually for each mouse.

### 2.6 Free AAs in Plasma, *M. quadriceps* Lysate, and Urine and Urinary N‐Metabolites

Plasma and acidified urine collected at d10 were diluted by factor 10 with ultra‐pure water and 40 mM phosphate buffer (pH 7.45), respectively. The free AAs in plasma and urine were analysed by HPLC on a Gemini 250 × 4.6 mm 5 μm C18 110 Å reverse‐phased column (Phenomenex, Aschaffenburg, Germany) [16]. The concentrations of urinary N‐metabolites were measured by HPLC as described [17]. One‐methyl‐histidine (1‐MH) and 3‐methyl‐histidine (3‐MH) concentrations in *M. quadriceps* tissue (30 mg) lysates (diluted by factor 2) were determined after homogenization by sonication and analysed as described above.

### 2.7 Clinical Chemistry, Plasma Metabolites and Proteins

Plasma activities of aspartate aminotransferase (ASAT), alanine aminotransferase (ALAT), alkaline phosphatase (AP), pseudocholinesterase (PCHE), and lipase, the plasma levels of albumin, glucose, bilirubin, cholesterol, triglycerides (TG), uric acid (UA), and urea, as well as Na, K, Ca, and Mg were determined using the chemistry analyser DxC 700 AU (Beckman Coulter, Krefeld, Germany) and systems reagents from Beckman Coulter for this analyser. Taurocholic acid (TCA), taurochenodeoxycholic acid (TCDCA), glycocholic acid (GCA), and cholic acid (CA) were assessed using the MxP Quant 500 kit (BIOCRATES Life Science AG, Innsbruck, Austria). Measurements were performed according to the manufacturer's instructions (for details see Supplemental Experimental Procedures).

ELISA assays were used to determine plasma levels of IGF‐1 (mouse IGF‐I; Mediagnost, Reutlingen, Germany), IGF‐1 binding protein (BP) 1 and 3 (mouse IGFBP‐1; mouse IGFBP‐3 (DuoSet Quantikine ELISA kit; R&D Systems Inc. Minneapolis, USA)), and myostatin (GDF‐8/Myostatin Quantikine ELISA kit (R&D Systems)) according to manufacturer's instructions. Plasma levels of epithelial growth factor (EGF) and its receptor EGFR‐1, hepatocyte growth factor (HGF‐1), fibroblast growth factor 21 (FGF‐21), interleukin 6 receptor alpha (IL‐6Rα), tumour necrosis factor receptor I (TNFRI) and TNFRII, and vascular endothelial growth factor (VEGF‐1) were determined with a bead‐based multiplex assay using the Luminex technology. Therefore, a mouse‐specific magnetic Luminex multiplex assay from Bio‐Techne (Minneapolis, MN, USA) was employed according to the instructions of the manufacturer. The Luminex 100/200 device was operated via the xPONENT 3.1‐Software (Luminex Corporate, Austin, USA).

### 2.8 Fractional Protein Synthesis Rate (FPSR) in Selected Organs

Thirty min before euthanasia, mice were injected i.p. with ^2^H_5_‐ring‐phenylalanine (^2^H_5_‐Phe; 375 mg/kg BW; 99.1 atom% ^2^H, Euriso‐Top GmbH, Saarbrücken, Germany), dissolved in 0.9% NaCl solution. The mice were euthanized as described above, plasma was collected, and liver, pancreas, and *M. quadriceps* were explanted, washed in 0.9% NaCl solution, snap‐frozen in liquid nitrogen, and stored at −80°C. The determination of the FPSR is described in the Supplemental Experimental Procedures section.

### 2.9 Gene Expression

Muscle RNA was isolated from at least 50 mg of tissue from *M. quadriceps* and *M. gastrocnemius*. The RNA was purified, quantified and its integrity was assessed according to Schregel et al 2022 [18]. Purified RNA (1 μg) was reverse transcribed into cDNA using the Sensi‐FAST cDNA Synthesis Kit (Bioline, Berlin, Germany) according to the manufacturer's instructions. Primers for the target genes and reference genes were purchased from IDT (Antwerp, Belgium; details are given in Table S2). cDNA samples were analysed with quantitative reverse transcriptase PCR (q‐RT PCR) using the LC 96 system (Roche Diagnostics, Mannheim, Germany). Samples were analysed in duplicate (plus five additional samples: two inter‐run calibrators, a no‐template, a no‐enzyme, and a water control). The mRNA expression values of target genes were normalized to four stable reference genes: *B2m*, *Polr2a*, *Rplp0*, and *Ppia*. The PCR efficiency and quantification cycle (Cq) values were then obtained for each sample using LinRegPCR v 2014.5. The normalization of target genes mRNA was performed using the qBASEplus software, taking into account amplification efficiencies, inter‐run variations, and normalization factors. All data was reported according to the Minimum Information for Publication of Quantitative Real‐Time PCR Experiments (MIQE) guidelines [19].

### 2.10 Statistics

The data was analysed using GraphPad Prism (GraphPad Software, version 9.4.1, San Diego, CA, USA). Normal distribution of the data was tested with the Kolmogorov–Smirnov test and the Shapiro–Wilk test. The sample sizes are given in the tables and legends of the figures, which also describe the presentation of the data and the statistical tests used for the data analysis. Adjusted *p*‐values of < 0.05 were considered statistically significant.

## 3 Results

### 3.1 BDL Causes CLD

**FIGURE 1:**
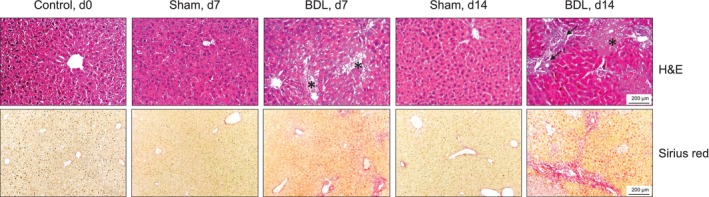
**H&E and Sirius red staining of liver tissue sections of mice with BDL or sham surgery and of controls** The mice were sacrificed on postoperative days 7 (d7) and 14 (d14). Mice without surgery (d0) served as controls. Formalin‐fixed paraffin‐embedded sections of liver tissue were deparaffinated and stained as indicated. Upper row: Asterisks point to necrotic tissue, and arrows to areas of inflammation, bile duct proliferation, and fibrosis. Lower row: Collagen fibres are coloured red. The findings are typical for 8 animals per time point. BDL–bile duct ligation, H&E–haematoxylin–eosin.

Consistent with previous studies in C57BL/6 mice, BDL, but not sham surgery, caused a severe CLD with extensive necrosis and inflammation. Furthermore, a beginning fibrosis was observed which progressed from postoperative days 7 to 14, as indicated by increased deposition of collagen (Figure 1).

As expected, CLD was associated with strongly elevated levels of bilirubin as well as ASAT‐, ALAT‐, and AP‐activities in plasma. We also noticed increased lipase activities in the BDL groups, compatible with a concomitant pancreatic response as a result of CLD (Table 1). Albumin and urea levels did not differ significantly between the sham and BDL groups, while PCHE was elevated after BDL. The reduced blood glucose and TG levels after BDL gave a first indication of the reduced nutritional status of these mice. Furthermore, BDL was associated with increased plasma levels of cholesterol and Ca (Table 1). The plasma concentrations of UA, Na, K, and Mg, as well as the urine concentrations of urea and UA, did not differ between the sham and BDL groups (data not shown).

**TABLE 1:** Blood plasma parameters of mice with BDL or sham surgery and of controls.

Parameter		Control	Sham	BDL	Sham	BDL
		d0	d7	d7	d14	d14
[Unit]		(*n* = 33 − 34)	(*n* = 12 − 20)	(*n* = 13 − 19)	(*n* = 19)	(*n* = 17)
Bilirubin	[μmol/L]	2.5 ± 0.2	1.4 ± 0.2	311.1 ± 17.0****	1.8 ± 0.2	317.7 ± 17****
ASAT	[U/L]	96.9 ± 6.5	87.3 ± 11.9	1677.2 ± 257.7****	73.6 ± 10.0	1217.7 ± 163.1****
ALAT	[U/L]	34.7 ± 2.9	39.1 ± 6.9	806.9 ± 107.1****	47.7 ± 13.1	594.1 ± 90.7****
PCHE	[kU/L]	3.5 ± 0.1	3.2 ± 0.1	5.1 ± 0.1****	3.1 ± 0.1	5.3 ± 0.2***
AP	[U/L]	78.8 ± 2.7	55.1 ± 2.3	966.2 ± 90.3****	60.9 ± 1.5	1623.4 ± 101.8****
Lipase	[U/L]	64.7 ± 2.8	51.9 ± 3.2	208.6 ± 36.7****	58.7 ± 3.1	449.2 ± 87.3****
Urea	[mmol/L]	9.9 ± 0.2	10.0 ± 0.3	20.6 ± 5.0	10.3 ± 0.3	13.1 ± 1.4
Albumin	[g/L]	28.1 ± 0.4	27.2 ± 0.6	29.0 ± 0.7	25.8 ± 0.3	26.4 ± 0.7
Glucose	[mmol/L]	13.4 ± 0.8	15.9 ± 1.3	8.2 ± 0.4****	14.8 ± 0.9	6.9 ± 0.4****
Cholesterol	[mmol/L]	2.2 ± 0.1	2.2 ± 0.1	17.9 ± 2.3****	2.1 ± 0.1	17.8 ± 1.9****
TG	[mmol/L]	1.0 ± 0.1	1.2 ± 0.1	0.8 ± 0.1*	1.0 ± 0.1	0.7 ± 0.1
Ca	[mmol/L]	2.2 ± 0.03	2.2 ± 0.06	2.6 ± 0.06***	2.2 ± 0.03	2.7 ± 0.04****

Parameter		Control	Sham	BDL	Sham	BDL
		d0	d7	d7	d14	d14
[Unit]		(*n* = 4 − 5)	(*n* = 4 − 6)	(*n* = 6)	(*n* = 5 − 6)	(*n* = 6)
TCA	[μmol/L]	0.9 ± 0.4	0.8 ± 0.1	225.5 ± 23.2*	9.3 ± 5.2	203.1 ± 23.7*
TCDCA	[μmol/L]	0.06 ± 0.02	0.05 ± 0.01	43.1 ± 5.1*	0.32 ± 0.16	28.3 ± 3.5
GCA	[μmol/L]	0.02 ± 0.00	0.03 ± 0.01	30.8 ± 7.4*	0.04 ± 0.01	11.2 ± 4.5
CA	[μmol/L]	0.11 ± 0.04	0.47 ± 0.08	0.084 ± 0.021*	2.7 ± 2.2	0.50 ± 0.14

*Note:* The mice were sacrificed on postoperative days 7 (d7) and 14 (d14). Mice without surgery (d0) served as controls. Data are presented as mean ± SEM. *p < 0.05, ***p < 0.001, ****p < 0.0001; BDL vs. sham‐operated mice; One‐way ANOVA with Šídák's multiple comparisons test (normally distributed data); Kruskal‐Wallis‐Test with Dunn's multiple comparisons test (not‐normally distributed data).

Abbreviations: BDL–bile duct ligation, ASAT–aspartate aminotransferase, ALAT–alanine aminotransferase, PCHE–pseudo‐cholinesterase, AP–alkaline phosphatase, TG–triglycerides, Ca–calcium, TCA–taurocholic acid, TCDCA–taurochenodeoxycholic acid, GCA–glycocholic acid, and CA–cholic acid.

Measurements of the plasma concentrations of individual bile acids showed a significant increase of TCA, TCDCA, and GCA after BDL, while the concentration of CA was diminished (Table 1).

### 3.2 CLD Is Associated With a Catabolic State and an Altered Protein Metabolism

**FIGURE 2:**
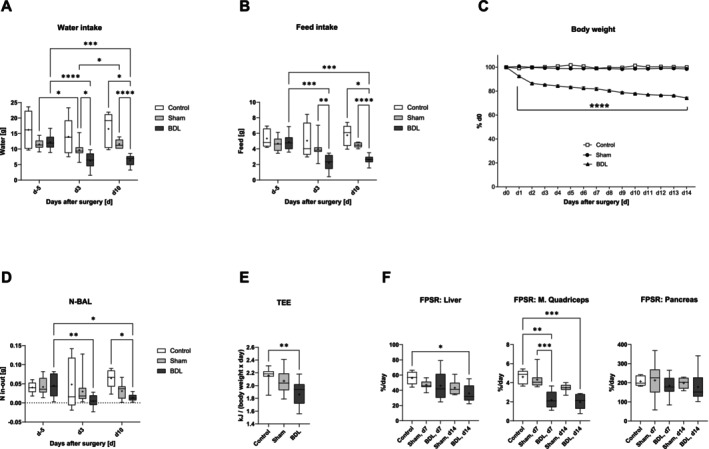
**Total water and feed intake, body weight, N‐BAL, TEE, and FPSR of mice with BDL or sham surgery and of controls**. The mice underwent BDL or sham surgery as indicated, or served as intact controls. Measurements were performed as described in the Methods section. TEE (total energy expenditure) was calculated from postoperative days 2 (d2) to d10. Data are shown as mean ± SEM (C), or as box plots (min‐max) with median (horizontal line) and mean (dot) (A, B, D–F). Number of mice (controls‐Sham‐BDL): 5–11‐10 in A, B, and D; 14–19‐17 (C); 8–11‐11 (E), and 5–6 (controls) and 8–9 (all other groups) (F). *p < 0.05, **p < 0.01, ***p < 0.001, ****p < 0.0001. Two‐way Repeated Measures (RM) ANOVA with Tukey‘s multiple comparisons test (A, B, D); mixed‐effects model with Geisser–Greenhouse correction and Tukey's multiple comparisons test (C); Kruskal‐Wallis‐Test with Dunn's multiple comparisons test (E and F; *M. quadriceps* ); one‐way ANOVA with Šídák's multiple comparisons test (F; liver and pancreas). In C, for a better overview, only the significant differences between the groups are shown, but not between the time points. BDL–bile duct ligation, N‐BAL–nitrogen balance, FPSR–fractional protein synthesis rate.

Mice with BDL‐mediated CLD showed reduced total water and feed intake, which was associated with significant body weight loss during the two‐week study period (Figure 2A–C). In contrast, there was only a transient decrease in water intake in mice with sham operations, most likely due to perioperative stress (Figure 2A). BDL also resulted in a lower N‐BAL, suggesting a loss of total body protein additionally related to lower protein intake (Figure 2D). In addition, lower TEE was observed in mice with BDL reflecting the lower feed intake (Figure 2E). Measurements of FPSR in mice with BDL revealed decreased rates of protein synthesis in the liver and *M. quadriceps* , while FPSR in the pancreas remained unaffected (Figure 2F). The effects of BDL compared to untreated controls, sham‐operated mice, or both were significant. In contrast, mice with sham surgery did not differ from controls (Figure 2A–F).

**FIGURE 3:**
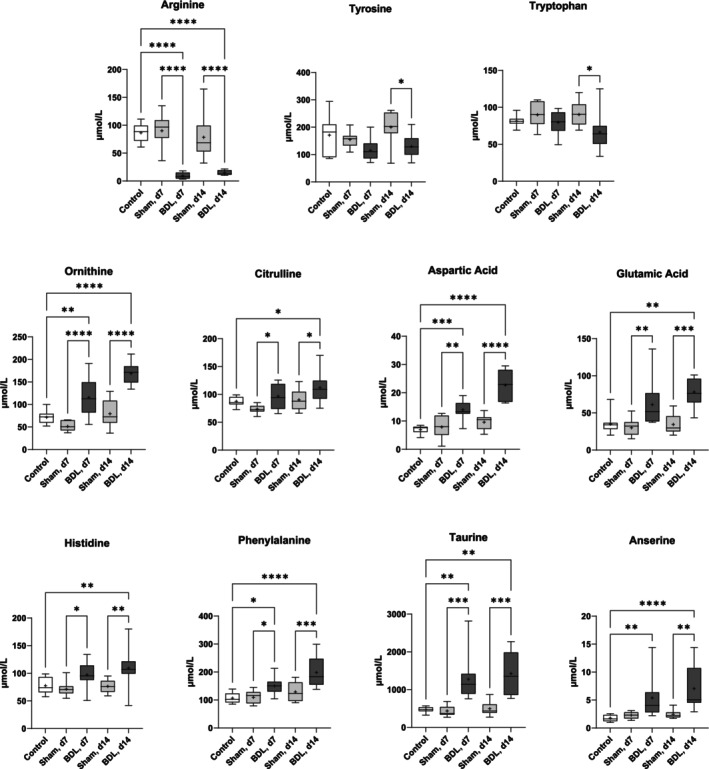
**Plasma free amino acids of mice with BDL or sham surgery and of controls.** The mice were sacrificed on d0 (controls), or postoperative days 7 (d7) and 14 (d14). Data are shown as box plots (min‐max) with median (horizontal line) and mean (dot). Number of mice: all groups: *n* ≥ 7. *p < 0.05, **p < 0.01, ***p < 0.001, ****p < 0.0001. One‐way ANOVA with Šídák's multiple comparisons test (arginine, ornithine, citrulline, aspartic acid, phenylalanine, tyrosine, and tryptophan); Kruskal‐Wallis‐test with Dunn's multiple comparisons test (all others). BDL–bile duct ligation.

To gain deeper insights into protein metabolism, plasma levels of AAs were determined (Figure 3). It is striking that only one AA, arginine, showed lower levels after BDL compared to both controls and sham treatment. In addition, tyrosine and tryptophan were selectively decreased at d14 in the comparison between the BDL and sham groups. Ornithine and citrulline, which like arginine are involved in urea synthesis, were increased after BDL, as were aspartic and glutamic acid, histidine, and phenylalanine. BDL was also associated with increased plasma levels of taurine, which is involved in bile acid conjugation and is found in high concentrations in muscles [20], and anserine (β‐alanyl‐N1‐methyl‐histidine), a dipeptide with antioxidative properties that is synthesized mainly in the muscles of animals but not in humans [21]. Plasma AAs that did not differ between mice with BDL and sham surgery are included in Table S1. The table also shows that neither of the families of essential nor non‐essential, branched‐chain, ketogenic, or glucogenic AAs displayed significant differences between these experimental groups.

### 3.3 Sarcopenia Is an Early Event in BDL‐Mediated CLD

Muscle energy expenditure is the most important contributor to resting energy expenditure, which in turn largely determines TEE [22]. Thus, both reduced FPSR in skeletal muscle (Figure 2F) and diminished TEE (Figure 2E) suggest that BDL plus the reduced feed intake triggers sarcopenia.

**FIGURE 4:**
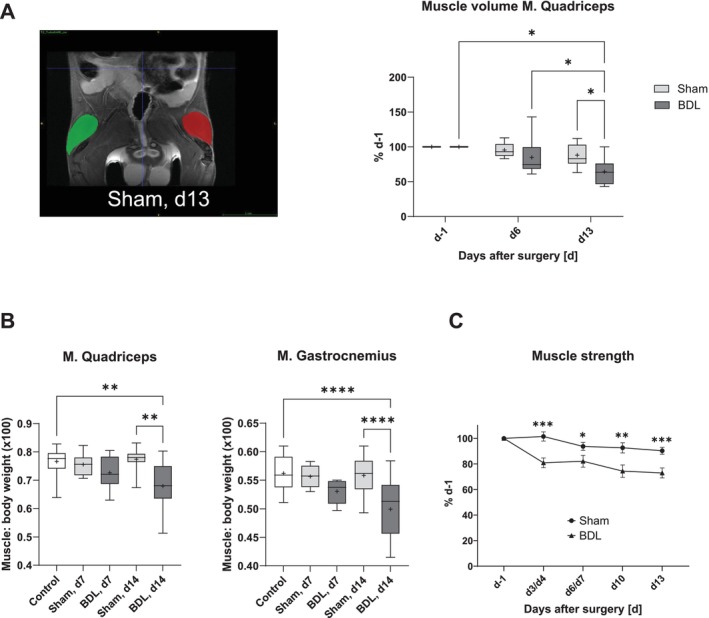
**Muscle volume, muscle‐to‐body weight ratio, and muscle strength of mice with BDL or sham surgery as well as intact controls**. Mice with BDL or sham surgery and intact controls underwent repeated measurements of muscle volume by MRI (A) and muscle strength using a grip strength meter (C). On days 7 and 14 after surgery, mice were sacrificed, and the muscle‐to‐body weight ratio was determined for the quadriceps and gastrocnemius muscles (B). Data in (A) are shown as box plots (min‐max) with median (horizontal line) and mean (dot). Number of mice: Sham: *n* = 11, BDL: *n* = 6. *p < 0.05 (two‐way RM ANOVA with Tukey‘s multiple comparisons test). On the left is an example T2‐weighted image of the analysis of the quadriceps muscle volume (coronal view, left (red) and right (green) muscles are marked). Data in (B) are presented as box plots (min‐max) with median (horizontal line) and mean (dot). Number of mice: *n* ≥ 6. **p < 0.01, ****p < 0.0001 (one‐way ANOVA with Šídák's multiple comparisons test ( *M. quadriceps* ); Kruskal‐Wallis‐test with Dunn's multiple comparisons test (*M. gastrocnemius*)). (C) Data are shown as mean ± SEM. Number of mice: Sham: *n* = 19, BDL: *n* = 18. *p < 0.05, **p < 0.01, ***p < 0.001 (two‐way RM ANOVA with Tukey‘s multiple comparisons test). For a better overview, only the significant differences between the groups are shown in (C), but not between the time points. BDL–bile duct ligation.

MRI measurements revealed that BDL, but not sham surgery, led to a decrease in the volume of the *M. quadriceps* over the investigation period of two weeks (Figure 4A). In addition, a lower skeletal muscle‐to‐body weight ratio was observed on day 14 after BDL, as shown by the weights of the quadriceps and gastrocnemius muscles (Figure 4B). Of note, CLD was also associated with reduced muscle strength, which was already detectable on the third day after BDL (Figure 4C).

**FIGURE 5:**
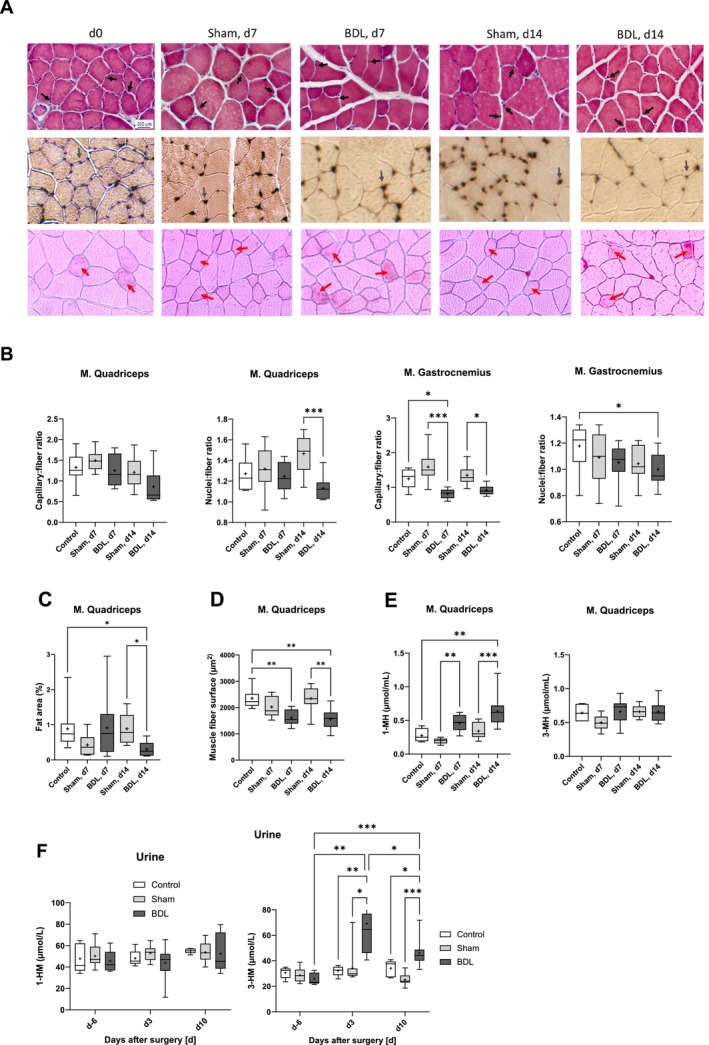
**Muscle histology and levels of methylhistidine in muscle and urine of mice with BDL or sham surgery and of controls** (A) (H&E, upper row), ammonium sulphide (middle row), and Oil Red O (lower row) staining of *M. quadriceps* tissue sections of mice with BDL or sham surgery and of controls. Mice were sacrificed on postoperative days 7 (d7) and 14 (d14). Mice without surgery (d0) served as controls. Frozen sections of *M. quadriceps* tissue were stained as indicated. Typical pictures for each group (control (*n* = 10), BDL (*n* = 9/10), and Sham (*n* = 8/9) and time point (d0, d7, and d14) are shown (original magnification x100)). Upper row: black arrows point to nuclei. Middle row: grey arrows point to capillaries. Lower row: red arrows point to muscle fibres with lipid droplets. (B) Nuclei, fibres, and capillaries in H&E sections of the quadriceps and gastrocnemius muscles were counted using the Olympus Cell^F software, and the capillary‐to‐fibre‐ and nuclei‐to‐fibre‐ratios were calculated. (C) Lipid droplets in Oil Red O sections of the quadriceps muscle were assessed using an Olympus BX43 microscope (UC30 camera (OSIS), CellSens software (Evident)). The area with fat droplets is expressed as a percentage of the total cross sectional area. (D) The muscle fibre surface was measured on H&E slides of the *M. quadriceps* using the muscle fibre measurement module of the CellSens software. (E and F) Concentrations of 1‐MH and 3‐MH were measured in muscle tissue lysates and repeatedly collected samples of urine, respectively. Data in (B‐F) are shown as box plots (min‐max) with median (horizontal line) and mean (dot). Number of mice: (B – D): *n* = 8–10; (E) controls: *n* = 4, other groups: *n* = 9; (F) controls: *n* = 5; Sham and BDL: *n* = 11. *p < 0.05, **p < 0.01, ***p < 0.001. One‐way ANOVA with Šídák's multiple comparisons test (Nuclei: fibre ratio *M. quadriceps* , 1‐MH and 3‐MH in muscle lysates); two‐way RM ANOVA with Tukey‘s multiple comparisons test (1‐MH and 3‐MH in urine); Kruskal‐Wallis‐test with Dunn's multiple comparisons test (all others). BDL–bile duct ligation.

We next analysed the histology of the quadriceps and gastrocnemius muscles. There was a general trend toward lower capillary‐to‐fibre and nuclei‐to‐fibre ratios in the BDL groups compared to sham‐operated mice and controls (Figure 5A,B). Additionally, the quadriceps muscle of BDL mice showed fewer lipid droplets within the muscle fibres after 14 days (Figure 5C) and a smaller muscle fibre surface after 7 and 14 days (Figure 5D) when compared to sham‐operated mice and untreated controls.

Interestingly, the concentrations of 1‐MH, a component of anserine, in the quadriceps muscle were significantly higher in mice with BDL than in the sham surgery and control groups, suggesting skeletal muscle damage. In contrast, levels of 3‐MH, a marker of a breakdown of the myosin and actin muscle proteins, did not differ between the experimental groups (Figure 5E). In urine, concentrations of 3‐MH but not of 1‐MH were significantly higher after BDL (Figure 5F). Taken together, the data shown in Figures 3, 4, 5 pointed to a state of muscle wasting and sarcopenia in mice with BDL.

**FIGURE 6:**
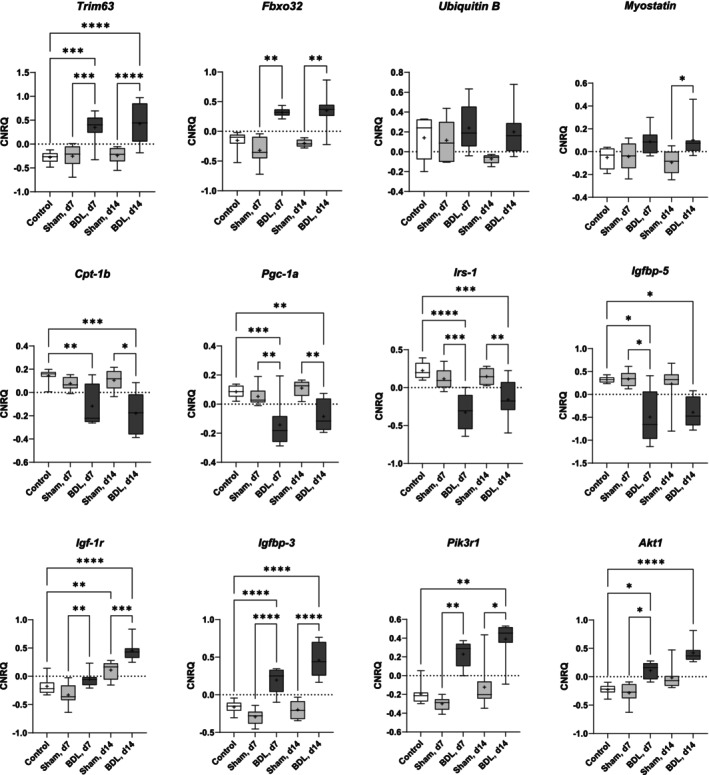
**Expression of anabolic and catabolic genes in the quadriceps muscle of mice with BDL or sham surgery and of controls** Mice underwent BDL or sham surgery and were sacrificed on postoperative days 7 (d7) and 14 (d14). Mice without surgery served as controls. Gene expression in the quadriceps muscle was quantified by real‐time PCR as described in the Methods section. Relative gene expression levels are expressed as CNRQ (calibrated normalized relative quantity) values. Data are shown as box plots (min‐max) with median (horizontal line) and mean (dot). Number of mice: *n* = 8. *p < 0.05, **p < 0.01, ***p < 0.001, ****p < 0.0001. One‐way ANOVA with Šídák's multiple comparisons test (*Trim63*, *Pgc‐1α*, *Irs‐1*, *Igf‐1r*, and *Igfbp‐3*); Kruskal‐Wallis‐Test with Dunn's multiple comparisons test (all other genes). BDL–bile duct ligation, *Trim63*–Tripartite Motif Containing 63, *Fbxo32*–Fbox protein 32, *Cpt‐1b*–carnitine palmitoyltransferase 1b, *Pgc‐1α*–peroxisome proliferative activated receptor, gamma, coactivator 1 alpha, *Irs‐1*–Insulin receptor substrate 1, *Igfbp‐3/5*–Insulin‐like growth factor 1‐binding protein 3/5, *Igf‐1r* –Insulin‐like growth factor 1 receptor, *Pik3r1*–Phosphatidylinositol 3‐kinase r1 subunit.

To gain mechanistic insights into muscle pathology, we analysed the expression of various genes in quadriceps muscle that are involved in anabolic and catabolic processes as well as mitochondrial function (Figure 6). Compared to controls without surgery and sham‐operated animals, mice with BDL had significantly or tended to have higher mRNA levels of genes associated with atrophy and protein degradation [23] (Tripartite Motif Containing 63 (*Trim63*), Fbox protein32 (*Fbxo32*), and Ubiquitin B) and the muscle growth inhibitor Myostatin (*Mstn*) [24]. In contrast, the mRNA levels of the mitochondrial enzyme carnitine palmitoyltransferase 1b (*Cpt1b*) and the key regulator of mitochondrial biogenesis and respiration peroxisome proliferative activated receptor, gamma, coactivator 1 alpha (*Pgc‐1α*; *Ppargc1α*) were reduced after BDL. Different expression profiles were observed for genes involved in the action of insulin and insulin‐like growth factors: While Insulin receptor substrate 1 (*Irs‐1*) and *Igfbp‐5* were downregulated, the expression of Igf‐1 receptor (*Igf‐1r*) and *Igfbp‐3* was higher in the BDL groups (days 7 and 14 after surgery, respectively). In addition, upregulation of Phosphatidylinositol 3‐kinase r1 subunit (*Pik3r1*) and *Akt1*, genes involved in the induction of muscle growth [25], was observed in these mice. Similar gene expression profiles were observed for the gastrocnemius muscle (Figure S1). Taken together, the gene expression profiles suggest that degenerative processes occur in the skeletal muscles of mice with BDL‐induced CLD, which in turn may induce compensatory counter‐regulation.

### 3.4 Mediators of Inflammation, Catabolic, and Anabolic Processes in Blood Plasma

**FIGURE 7:**
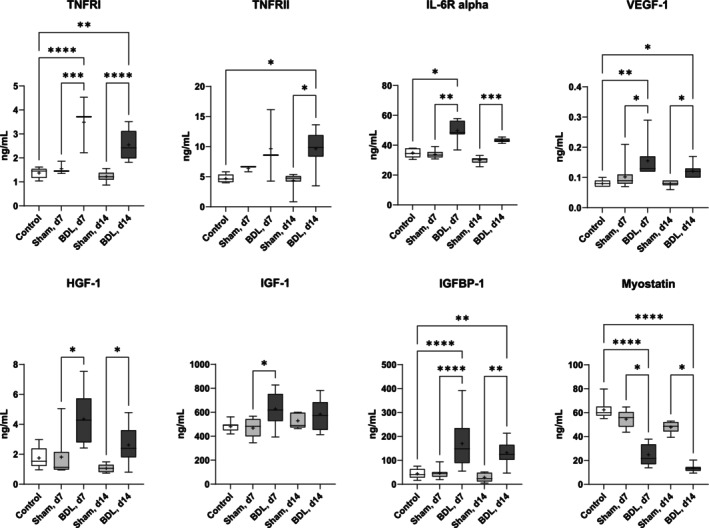
**Plasma protein concentrations of mediators of inflammation, catabolic, and anabolic processes of mice with BDL or sham surgery and of controls** Mice with BDL or sham surgery were sacrificed on postoperative days 7 (d7) and 14 (d14). Mice without surgery served as controls. The plasma protein levels were determined by Luminex assays and ELISA, respectively, as described in the Methods section. Data are shown as box plots (min‐max) with median (horizontal line) and mean (dot). Number of mice: *n* ≥ 7 (TNFRI/II: control *n* = 5; Sham, d7 and BDL, d7 *n* = 3; Sham, d14 and BDL, d14 *n* = 9). *p < 0.05, **p < 0.01, ***p < 0.001, ****p < 0.0001. One‐way ANOVA with Šídák's multiple comparisons test (TNFRI, IGFBP‐1); Kruskal‐Wallis‐Test with Dunn's multiple comparisons test (all other proteins). BDL–bile duct ligation, TNFRI/II–tumor necrosis factor I/II, IL‐6R – interleukin 6 receptor, VEGF‐1–Vascular endothelial growth factor 1, HGF‐1–Hepatocyte growth factor 1, IGF‐1–insulin‐like growth factor 1, IGFBP‐1–IGF‐1 binding protein 1.

In mice with BDL, significantly higher plasma protein concentrations of TNFRI, TNFRII, and IL‐6Rα than in sham‐operated mice and intact controls were observed; results that are consistent with a systemic pro‐inflammatory state associated with CLD. BDL mice also showed higher levels of VEGF‐1 (which is involved in vascular growth, but also in liver fibrosis [26]) and the mediator of liver regeneration HGF‐1 [27]. Strikingly, the anabolic hormone IGF‐1 [25] and its binding protein IGFBP‐1 were also elevated, while levels of the myokine myostatin were *reduced* after BDL (Figure 7). No differences between mice with BDL and sham surgery were observed for the hepatokine FGF‐21, IGFBP‐3, EGF‐1, and EGFR‐1 (Figure S2).

## 4 Discussion

Disease‐related malnutrition and sarcopenia are characteristic features of patients with end‐stage liver disease and influence the course of the disease unfavourably. Notwithstanding the prognostic significance of malnutrition and sarcopenia in LC, diagnosis is difficult and the exact pathophysiological mechanisms are not fully understood [8]. Last but not least, there is a lack of validated biomarkers for early diagnosis. Unlike studies in patients, animal studies offer the advantage that the development of malnutrition and sarcopenia can be investigated over the entire time course and from the earliest point of liver injury.

In this study, we focused on early changes in metabolism and muscle function associated with CLD using the model of BDL in mice. Liver histology and basic laboratory findings indicated that BDL caused a severe cholestatic liver injury. CLD progressed over the investigation period of 14 days to a stage with liver cell necroses, inflammation, and beginning fibrosis but still without a generalized failure of liver synthesis functions as a reduced rate of hepatic protein synthesis only becomes apparent 14 days after surgery. Progressive body weight loss, lower intake of water and feed, a lower N‐BAL, and reduced TEE pointed to a catabolic state of BDL mice. The lower TEE value could be explained by the lower energy requirement to metabolize the lower amount of feed ingested and by the lower energy requirement to maintain and move a lighter body [28]. However, after surgery, TEE decreased by about 20% in the BDL mice, which was related to the approximately 20% body weight loss on d3 and d10, but not to the 50% decrease in feed intake. Given that resting energy expenditure is the parameter that mostly affects TEE and that muscle energy expenditure is the primary contributor to resting energy expenditure [22], it is plausible that the weight loss of BDL mice is primarily a loss of metabolically active tissue, such as skeletal muscle–which was also detected by MRI. Interestingly, while feed and, therefore, protein intake was reduced by approximately 50% of the intake prior to BDL surgery, N‐BAL was reduced by 87% at d10 compared to the N‐BAL observed in healthy mice before surgery. The lower N‐BAL is likely related to a disproportionately greater N loss caused by the enhanced protein catabolism associated with inflammation in liver disease [29], as suggested by the increased plasma levels of IL‐6Rα, TNFRI, and TNFRII in BDL mice. The reduced feed/protein intake and the pro‐inflammatory state of the BDL mice are likely to be major contributors to the reduced rates of protein synthesis in the liver and skeletal muscle, potentially exacerbating the lower N‐BAL.

Mice with BDL develop sarcopenia as evidenced by decreased muscle strength, muscle volume, and muscle‐to‐body weight ratio. Histological signs of muscle atrophy observed in the BDL groups were lower capillary‐to‐fibre and nuclei‐to‐fibre ratios and reduced muscle fibre surfaces. It has been shown that liver damage may lead to myosteatosis, which in turn negatively affects muscle function [30]. On the other hand, it is known from human studies that a catabolic state, as exists in the BDL mice, can cause lipolysis in the skeletal muscles in order to mobilize fat sources as energy for the body [31].

In addition, the gene expression profile of the skeletal muscles examined (with increased expression of genes associated with atrophy and protein degradation) and the increased levels of 1‐MH in the quadriceps muscle and 3‐MH in the urine indicate progressive muscle wasting. A key finding of this study is that sarcopenia occurs very early in the course of the CLD‐promptly after liver damage and not only after a latency period in which malnutrition first develops. In our search for the causal factors, we focused our attention on circulating metabolites and hormones.

Plasma levels of most AAs remained remarkably constant after BDL. In contrast to what is known for decompensated LC [7], the levels of aromatic and branched‐chain amino acids did not change significantly, and the concentrations of essential, non‐essential, ketogenic, and glucogenic AAs remained unchanged as well. However, the following specific changes in AA and nitrogen metabolism stand out: First, BDL was associated with strikingly elevated plasma taurine levels, which may be due to a combination of underutilization for bile acid conjugation and release from damaged hepatocytes [32] and skeletal muscle, where taurine is the most abundant free AA and essential for contraction [20]. The release from injured muscle tissue is probably also the cause of the increased anserine, 1‐MH and histidine concentrations after BDL. Secondly, opposite changes were observed in the AAs of the urea cycle, with increased concentrations of ornithine and (to a much lesser extent) citrulline, but greatly reduced arginine levels. Low arginine concentrations after BDL in rats have previously been linked to high circulating levels of arginase, which would also explain the elevated ornithine levels [33]. The conspicuously low plasma concentration of arginine in BDL also deserves attention concerning muscle performance. First of all, arginine is an amidine donor for guanidoacetic acid and subsequent creatine synthesis and therefore required for the primary storage energy phosphagen, creatine phosphate [34]. Moreover, arginine serves as a substrate for nitric oxide synthase (NOS) to produce nitric oxide (NO). Skeletal muscle functions regulated by NO or related molecules include excitation‐contraction coupling, regulation of blood flow, myocyte differentiation, respiration, and glucose homeostasis [35].

At the level of lipid metabolism, an increase in cholesterol and a decrease in TG in plasma after BDL were observed. The former could be due to increased release from damaged hepatocytes [36], while the latter could primarily be the result of an absorption disorder due to a lack of bile acids in the intestine and reduced micelle formation [37]. It is noteworthy that the plasma glucose concentration decreased significantly after BDL. Possible causes are reduced gluconeogenesis in the damaged liver and reduced glycogen availability, which is probably related to malnutrition. Overall, the analyses of plasma metabolites suggested a reduced availability of substrates for muscle energy production, which may impair skeletal muscle function. Elevated calcium levels, as observed in this study, could act as an aggravating factor.

The investigations of hepatokines, myokines, and other hormonal mediators yielded results that should be evaluated differently. Some findings in BDL mice, such as the increased concentrations of HGF‐1, IGF, IFGBP‐1, and the unexpectedly low plasma level of the myokine myostatin (despite increased *Myostatin* mRNA in the quadriceps muscle), are potentially consistent with processes of counter‐regulation and regeneration after the initial liver injury and the resulting muscle wasting. However, it was also shown that there is a progressive decrease in myostatin levels as cirrhosis progresses [38]. The expression profiles of some genes in the skeletal muscles of BDL mice, such as the increased mRNA levels of *Pik3r1*, *Akt*, *Irs‐1*, and *Igfbp‐3*, would also be compatible with ongoing (although insufficient) repair processes. This is opposed by the increase of IL‐6Rα, TNFRI, and TNFII in the blood plasma of BDL mice, indicating a systemic pro‐inflammatory state that promotes sarcopenia.

As was to be expected after BDL, there was a sharp increase in conjugated bile acids in the blood plasma. Importantly, bile acids have been shown to promote sarcopenia under conditions of chronic liver disease. Bile acid action in muscle cells has been shown to be mediated by the Takeda G protein‐coupled receptor 5 (TGR5) [39] and involves the induction of mitochondrial dysfunction [40]. While *Tgr5* mRNA levels were below the detection limit of our assay (data not shown), levels of *Cpt1b* and *Pgc‐1α* were significantly reduced after BDL. The impairment of mitochondrial function needs to be further validated in follow‐up studies.

In summary, the early development of sarcopenia after BDL is a multicausal process with reduced protein synthesis, increased muscle protein breakdown, low arginine levels (possibly related to nitrosative stress), a systemic pro‐inflammatory and catabolic state, and bile acid muscle toxicity as essential elements. Of course, the most efficient treatment of CLD and CLD‐associated sarcopenia is eliminating the cause. If this is not possible, as is often the case with CLD in humans, symptomatic measures such as anti‐inflammatory treatment, lowering bile acid levels and NOS activity with medication, targeted compensation of deficits, and physical exercise can offer the best chances of success in the treatment of sarcopenia.

## Conflicts of Interest

The authors declare no conflicts of interest.

## Supporting information


**Table S1** The concentrations of free plasma amino acids and anserine in mice with BDL or sham surgery, and in controls.
**Table S2** Technical details of gene expression analysis by qPCR.
**Figure S1** Effects of BDL on the expression of anabolic and catabolic genes in the gastrocnemius muscle. Mice underwent BDL or sham surgery and were sacrificed on postoperative days 7 (d7) and 14 (d14). Mice without surgery served as controls. Gene expression in the gastrocnemius muscle was quantified by real‐time PCR as described in the Methods section. Relative gene expression levels are expressed as CNRQ (calibrated normalized relative quantity) values. Data are shown as box plots (min‐max) with median (horizontal line) and mean (dot). *n* = 8 mice per group; * *p* < 0.05, ** *p* < 0.01, *** *p* < 0.001, **** *p* < 0.0001. One‐way ANOVA with Šídák’s multiple comparisons test ( *Ubiquitin B* , *Myostatin* , *Pgc‐1α, Insuline receptor substrate 1* ( *Irs‐1* ), *Insulin‐like growth factor binding protein 5* ( *Igfbp‐5* ), *Akt1* , *Phosphatidylinositol 3‐kinase r1 subunit* ( *Pik3r1* )); Kruskal‐Wallis‐Test with Dunn’s multiple comparisons test (all other genes).
**Figure S2** Plasma protein concentrations of mediators of inflammation, catabolic processes, and anabolic processes. Mice with BDL or sham surgery were sacrificed on postoperative days 7 (d7) and 14 (d14). Mice without surgery served as controls. The plasma protein levels were determined by Luminex assays and ELISA, respectively, as described in the Methods section. Data are shown as box plots (min‐max) with median (horizontal line) and mean (dot). *n* ≥ 7 mice per group (control *n* = 5); * *p* < 0.05. One‐way ANOVA with Šídák’s multiple comparisons test (FGF‐21); Kruskal‐Wallis‐Test with Dunn’s multiple comparisons test (all other proteins).
