# Supplementary material for: Changes in Protein Metabolism and Early Development of Sarcopenia in Mice With Cholestatic Liver Disease
Source: J Cachexia Sarcopenia Muscle. 2025 Feb 19;16(1):e13737. doi: 10.1002/jcsm.13737 (PMC11839266; doi:10.1002/jcsm.13737)
Supplement: Supplementary file 1 — Table S1 The concentrations of free plasma amino acids and anserine in mice with BDL or sham surgery, and in controls. Table S2 Technical details of gene expression analysis by qPCR. Figure S1 Effects of BDL on the expression of anabolic and catabolic genes in the gastrocnemius muscle. Mice underwent BDL or sham surgery and were sacrificed on postoperative days 7 (d7) and 14 (d14). Mice without surgery served as controls. Gene expression in the gastrocnemius muscle was quantified by real‐time PCR as described in the Methods section. Relative gene expression levels are expressed as CNRQ (calibrated normalized relative quantity) values. Data are shown as box plots (min‐max) with median (horizontal line) and mean (dot). n = 8 mice per group; * p < 0.05, ** p < 0.01, *** p < 0.001, **** p < 0.0001. One‐way ANOVA with Šídák’s multiple comparisons test ( Ubiquitin B , Myostatin , Pgc‐1α, Insuline receptor substrate 1 ( Irs‐1 ), Insulin‐like growth factor binding protein 5 ( Igfbp‐5 ), Akt1 , Phosphatidylinositol 3‐kinase r1 subunit ( Pik3r1 )); Kruskal‐Wallis‐Test with Dunn’s multiple comparisons test (all other genes). Figure S2 Plasma protein concentrations of mediators of inflammation, catabolic processes, and anabolic processes. Mice with BDL or sham surgery were sacrificed on postoperative days 7 (d7) and 14 (d14). Mice without surgery served as controls. The plasma protein levels were determined by Luminex assays and ELISA, respectively, as described in the Methods section. Data are shown as box plots (min‐max) with median (horizontal line) and mean (dot). n ≥ 7 mice per group (control n = 5); * p < 0.05. One‐way ANOVA with Šídák’s multiple comparisons test (FGF‐21); Kruskal‐Wallis‐Test with Dunn’s multiple comparisons test (all other proteins). [file JCSM-16-e13737-s001.pdf]

Supplemental Experimental Procedures

Magnetic resonance imaging (MRI) measurements of the M. quadriceps

The volumes of the left and right M. quadriceps were determined in anesthetized mice (1.5-2.5 % isoflurane in oxygen). The respiratory rate (maintained between 35 and 50 breaths/min) and body temperature of the animals were continuously monitored during the scan (respiration-triggered).

MRI was performed with a 7 Tesla MRI (Bruker BioSpec 70/30, gradient system: BGA 12 S HP, 86 mm volume coil in transmit mode, 2 x 2 receive surface coil, all Bruker Biospin GmbH, Ettlingen, Germany) using coronal T1 weighted FLASH sequences and T2 weighted TurboRARE sequences. Additional coronal and sagittal slice fractions were scanned and used for better anatomical orientation during muscle segmentation.

T1w FLASH (cor): TR: 313 ms, TE: 2.9 ms, field of view: 32.0 x 35.2 mm, matrix size: 256 x 281, in-plane resolution: 125 x 125 µm, FA: 50°, 4 averages, slice thickness: 0.8 mm, 23 to 26 slices depending on the size of the mouse, no slice gap, TA: approx. 5:50 min depending on the respiration rate and consequent trigger.

T2w TurboRARE (cor): TR: 1968 ms, TE: 25 ms, field of view: 32.0 x 35.2 mm, matrix size: 256 x 281, in-plane resolution: 125 x 125 µm, RARE factor: 8, 4 averages, slice thickness: 0.8 mm, 23 to 26 slices, no slice gap, TA: approx. 4:40 min depending on the respiration rate and consequent trigger.

Images were analyzed using ITK-Snap software (version 3.8.0, Penn Image Computing and Science Laboratory "PICSL", University of Pennsylvania, USA). Volume evaluation was based on a slice-wise independent placement of regions of interest in each muscle using a T2-weighted image series of coronal slices. Muscle volumes on both sides were scored in mm^3, and the average was calculated and related to d-1.

Total energy expenditure (TEE) and nitrogen balance (N-BAL) experiments in metabolic cages

Before and after BDL or sham surgery, mice were individually placed in metabolic cages (MC) for 24 h at the time points indicated in the manuscript. During the three MC trials, feed and total water intake (drinking water plus water contained in feed mixture) as well as body weight were monitored, and urine and feces were collected for each mouse individually.

On d2 after surgery, mice were injected i.p. with 8 mg/g body weight (BW) ^2H_2O (99.8 atom% ^2H, Chemotrade, Leipzig, Germany) plus 12 mg/g BW $H_2{}^{18}O$ (97 atom% ^{18}O, Eurisotop, Paris, France), sterilized by filtration through a 0.2 µm sterilized NALGENE® 4 mm nylon syringe filter (Thermo Scientific, Fisher Scientific GmbH, Schwerte, Germany) to determine TEE. Blood was collected on d10 and mice were euthanized as described in the manuscript.

Urine was collected over 24 h in three different tubes: a 15 mL tube, containing 150 µL of 50% sulfuric acid to prevent the loss of volatile N compounds and thus to be used to calculate N-BAL, N-excretion, and urinary AA, and two 2 mL tubes containing no acid at two different time points from 1.5 to 2.5 and from 22.5 to 24 h after the start of the MC trial, to be used for the measurement of ^{2}H and ^{18}O enrichment and to calculate TEE.

The TEE was assessed by isotope dilution using the doubly labeled water method.[1] The isotope ratios $^{2}H/^{1}H$ and $^{18}O/^{16}O$ (assessed on d2/3, d9/10) corrected for isotopic background (assessed on d-6/-5) in the non-acidified urine samples and the plasma samples collected on d10 were analyzed by gas isotope ratio mass spectrometry (DELTA Plus XL, Thermo Quest, Bremen, Germany) coupled with a gas bench (GasBench II, Finnigan, Bremen, Germany).[2] Samples of d2/3 had to be diluted with non-enriched standard water by factor 50 and 25, respectively. Tracer enrichments in plasma samples from d10 were equivalent to that of the 22.5 – 24 h urine samples collected at d10. If a d10 22.5 - 24 h urine sample could not be collected, the d10 plasma enrichment was used for TEE determination instead. The production of CO_2 (rCO_2) was calculated using the total body water pool (W) and the rate constants for ^{18}O (k_o) and ^{2}H (k_h) excretion:[3]

$$rCO_2\ (\text{mol } CO_2/d) = [(W/2078)\ (1.01\ k_o - 1.04\ k_h)] - [0.0246\ (1.05W\ (1.01\ k_o - 1.04\ k_h))]$$

The rCO_2 (converted into l/d) was used to calculate the TEE according to the Brouwer equation,[3,4] using a respiratory quotient (RQ) of 0.94 (mean RQ value taken from Guidotti et al[1]) and taking into account the urinary N loss (NU, in g/d) as follows:

$$TEE\ (kJ/(g*d)) = 5.16\ rCO_2 + 16.18\ rCO_2/\ RQ - 5.90\ NU$$

To calculate the N-BAL, the dry matter of feed, urine, and feces was determined by drying the samples at 55 °C (urine and feed overnight, feces 24 h) and measuring the N content in % of dry matter with an elemental analyzer (Thermo-Fisher Scientific GmbH, Waltham, MA, USA) in pre-dried tin capsules (IVA-Analysentechnik GmbH und Co. KG, Meerbusch, Germany). Subsequently, the 24 h N-BAL was calculated from the N intake and output, respectively, of feed (N_{feed}), urine (N_{urine}), and feces (N_{feces}) as follows:

$$N\text{-}BAL\ (g) = N_{feed} - (N_{urine} + N_{feces})$$

Fractional protein synthesis rate (FPSR)

The mice were injected i.p. with $^{2}H_5$-ring-phenylalanine ($^{2}H_5$-Phe), and tissues were harvested and stored as described in the manuscript.

Fifty mg of tissue were derivatized with N-methyl-N-tert-butyldimethylsilyltrifluoroacetamide (Acros Organics, Thermo Fisher Scientific GmbH, Geel, Belgium) as described[5] to determine the enrichment of protein-bound $^{2}H_5$-Phe. To measure the enrichment of free $^{2}H_5$-Phe in the precursor pool, 25 µL plasma was precipitated using acetonitrile and centrifuged (16,000 × g,

4°C 20 min). The supernatant was dried under N_2 and derivatized with N-methyl-N-tert-butyldimethylsilyltrifluoroacetamide. Both protein pellet and plasma $^2H_5$-Phe enrichments were determined in tert-butyldimethylsilyl derivatives using gas chromatography-mass spectrometry[5]. Diagnostic ions were m/z 234 (M+0) and m/z 239 (M+5).[6] For the muscle analyses, the signal of the m/z 239 was too low and the resolution could not be appropriately increased without overloading the system, thus the method was modified according to Welle et al (2006) (using m/z 235 (M+1) instead of m/z 237 (M+3)).[7]
The FPSR was determined according to Marini and Didelija (2015):[8]

$$\text{FPSR (\%/d)} = [E_{\text{protein-bound}}\,(\text{MPE})/E_{\text{free}}(\text{MPE})/t] \times 100$$

Where $E_{\text{protein-bound}}$ is the enrichment of $^2H_5$-Phe bound in tissue protein (product), E_{free} is the enrichment of the free $^2H_5$-Phe in the plasma (precursor), and t is the time in min from injection to sampling.

Assessment of bile acids using targeted LC-MS/MS profiling

Metabolomics measurements

Targeted metabolomics profiling of the plasma samples was performed using the MxP® Quant 500 kit (BIOCRATES LifeSciences AG, Innsbruck, Austria) at the Institute of Clinical Chemistry and Laboratory Medicine of the University Medicine Greifswald. 10 µL aliquots of each plasma sample were processed as recommended by the manufacturer. The fully automated assay combined flow injection (FIA) and LC-MS/MS selective detection. MS analyses were performed on an AB SCIEX 5500 QTrap™ mass spectrometer (AB SCIEX, Darmstadt, Germany) with electrospray ionization combined with a HPLC system (Agilent 1260 Infinity Binary LC, Santa Clara, United States) including a degasser unit, column oven, autosampler and a binary pump. Internal standards (isotope labelled) are partially integrated in the kit plate for metabolite quantification. After the measurements, the data are uploaded to the Biocrates MetIDQ software (part of the kit), where in a first pre-processing step, the peak integration and the calibration curves are checked before the automatic calculation of the metabolite concentrations is performed.

Metabolomics measurements: quality control and normalization of metabolite levels

To account for day-to-day variation in performance of the LC-MS/MS platform, we added up to three replicates of a pooled plasma sample obtained from healthy volunteers to each plate, i.e. measurement batch. The corresponding metabolite concentrations were used for normalization. For each plate the measured concentrations of the metabolites were divided by the median concentration of the pooled samples. Subsequently, for each metabolite the median of the plate-specific medians of the pooled samples was calculated to reset the concentration to the original scale (µM concentrations). For each metabolite the plate-specific

coefficient of variation (CV) was calculated based on the pooled samples. Only metabolites with at least a CV < 25% on two plates were included in the final data sets.

References

[1] Guidotti S, Meijer HAJ, van Dijk G. Validity of the doubly labeled water method for estimating CO2 production in mice under different nutritional conditions. American journal of physiology. Endocrinology and metabolism 2013;305:E317-24.

[2] Junghans P, Görs S, Langhammer M, Metges CC. Breath water-based doubly labelled water method for the noninvasive determination of CO2 production and energy expenditure in mice. Isotopes in environmental and health studies 2018;54:561–72.

[3] Lifson N. Theory of use of the turnover rates of body water for measuring energy and material balance. Journal of theoretical biology 1966;12:46–74.

[4] Blaxter KL, editor. Energy metabolism;: Proceedings of the 3rd symposium held at Troon, Scotland, May 1964. European Association for Animal Production. Publication, no. 11. London, New York: Academic Press; 1965.

[5] Schregel J, Schulze Holthausen J, Sciascia QL, Li Z, Görs S, Eggert A, et al. Effects of oral glutamine supplementation on jejunal morphology, development, and amino acid profiles in male low birth weight suckling piglets. PloS one 2022;17:e0267357.

[6] Dänicke S, Goyarts T, Döll S, Grove N, Spolders M, Flachowsky G. Effects of the Fusarium toxin deoxynivalenol on tissue protein synthesis in pigs. Toxicology letters 2006;165:297–311.

[7] Welle S, Bhatt K, Pinkert CA. Myofibrillar protein synthesis in myostatin-deficient mice. American journal of physiology. Endocrinology and metabolism 2006;290:E409-15.

[8] Marini JC, Didelija IC. Arginine depletion by arginine deiminase does not affect whole protein metabolism or muscle fractional protein synthesis rate in mice. PloS one 2015;10:e0119801.

Table S1 Concentrations of free plasma amino acids and anserine in mice with BDL or sham surgery and in controls

Amino Acid/	d0		Sham, d7		BDL, d7		Sham, d14		BDL, 14		BDL, d7 vs.	BDL, d14 vs.
Dipeptide (µmol/L)	Mean	SEM	Mean	SEM	Mean	SEM	Mean	SEM	Mean	SEM	Sham, d7	Sham, d14
Leucine	129,4	5,73	115,9	7,54	117,2	9,87	139,9	13,16	120,6	8,22		
Isoleucine	88,18	3,08	76,17	4,12	67,03	5,78	92,48	8,24	69,52	5,73		
Valine	231,0	6,22	192,4	6,81	176,8	12,40	239,8	18,05	195,0	14,64		
Lysine	225,2	7,07	187,3	7,05	201,4	16,00	219,0	16,64	237,3	11,10		
Methionine	52,40	1,51	46,47	2,99	51,94	3,70	55,72	2,77	64,16	3,65		
Cysteine	110,3	4,09	109,6	5,36	110,0	8,41	127,8	6,22	111,9	11,37		
Phenylalanine	106,7	5,35	109,6	6,58	149,9	9,52	128,9	9,86	189,5	16,52	p<0.05	p<0.001
Tyrosine	171,0	20,18	154,7	7,65	114,9	11,38	200,1	17,03	120,6	12,42		p<0.05
Threonine	184,9	4,19	166,2	9,69	166,0	10,13	198,0	8,93	199,5	13,49		
Tryptophan	81,45	2,09	90,03	5,17	79,71	4,73	90,30	4,93	59,86	7,50		p<0.05
Histidine	77,61	4,44	71,50	3,87	97,26	6,69	76,38	3,40	101,7	10,22	p<0.05	p<0.01
Arginine	86,58	4,83	90,19	7,70	9,31	1,87	78,40	11,61	15,00	1,52	p<0.0001	p<0.0001
Glutamine	631,7	23,68	618,5	32,68	769,1	50,64	711,0	28,02	854,9	49,81		
Glycine	267,6	10,47	263,1	16,74	275,4	23,00	298,1	7,97	317,5	21,34		
Proline	102,5	10,17	146,8	17,84	172,9	14,69	203,1	18,57	228,2	32,20		
Alanine	505,4	19,83	487,1	24,84	530,3	40,12	593,9	38,50	584,4	38,45		
Aspartic acid	6,96	0,40	7,87	1,12	14,03	0,99	9,62	0,79	22,08	1,63	p<0.01	p<0.0001
Asparagine	38,49	1,18	40,63	3,64	55,13	4,81	48,95	2,38	65,66	3,51		
Glutamic acid	35,01	3,84	29,99	3,24	61,18	9,15	34,43	3,78	76,01	5,63	p<0.01	p<0.001
Serine	134,8	7,36	128,2	6,19	149,0	10,59	151,8	5,23	176,4	7,72		
Citrulline	87,44	2,43	73,60	2,06	96,52	6,62	90,36	5,77	112,1	8,73	p<0.05	p<0.05
Ornithine	71,60	4,28	51,11	3,10	115,4	12,42	79,38	8,59	164,5	7,32	p<0.0001	p<0.0001
Hydroxyproline	13,00	1,65	24,93	3,93	34,43	4,58	35,16	4,21	39,10	4,81		
Taurine	475,9	22,04	432,5	40,39	1277	181,7	494,1	55,64	1385	167,7	p<0.001	p<0.001
Anserine	1,77	0,17	2,31	0,16	5,39	1,31	2,43	0,21	7,04	1,33		p<0.01
Essential amino acids	1263	28,33	1146	52,01	1115	62,71	1319	78,32	1249	81,63		
Non essential amino acids	1999	65,12	1987	92,58	2251	147,8	2379	94,96	2558	155,1		
Branched-chain amino acids	448,6	13,94	384,5	17,98	361,0	27,35	472,2	38,71	385,1	28,07		
Aromatic amino acids	436,8	26,69	425,8	16,49	441,8	24,82	495,7	29,87	471,6	41,63		
Gln family	855,7	32,57	885,5	50,77	1011	70,02	1027	37,23	1171	79,72		
Arg family	1056	33,02	1059	54,84	1290	87,76	1255	50,01	1535	83,68		p<0.01
Ketogenic amino acids	354,7	10,10	303,2	13,84	318,6	23,26	359,0	27,78	357,9	18,43		
Glucogenic amino acids	907,8	31,60	878,5	44,53	954,8	71,51	1044	49,90	1078	64,28		

Note The mice were sacrificed on postoperative days 7 (d7) and 14 (d14). Mice without surgery (d0) served as controls. Data are shown as mean ± SEM. One-way ANOVA with Šídák's multiple comparisons test (normally distributed data); Kruskal-Wallis-Test with Dunn's multiple comparisons test (not-normally distributed data). Abbreviations: BDL: Bile duct ligation surgery group; Sham: Sham surgery group.

Table S2 Gene expression analysis by qPCR – technical details

IGF-1 signalling genes						
**Gene Name**	**Gene Abbreviation**	**PrimeTime qPCR Primer Assay**	**Sequence F/R**	**Tissue**	**PCR Efficiency**	**Quantification cycle value (Cq)**
Insulin Like Growth Factor 1 Receptor	*Igf1r*	Mm.PT.58.11619137	5‘-GCCTACCTCAATGCCAACA-3‘	*M. gastrocnemius*	2.03	28
			5‘-TCTCGTAGATGTCTCGTGTCA-3‘	*M. quadriceps*	2.03	29
Insulin Like Growth Factor Binding Protein 3	*Igfbp3*	Mm.PT.58.6744601	5‘-CATCTGAAGTTCCTCAATGTGC-3‘	*M. gastrocnemius*	2.13	28
			5‘-CCATACTTGTCCACACACCA-3‘	*M. quadriceps*	2.16	28
Insulin Like Growth Factor Binding Protein 5	*Igfbp5*	Mm.PT.58.11593699	5‘-GTACCTGCCCAACTGTGAC-3‘	*M. gastrocnemius*	2.11	25
			5‘-GCTTCATTCCGTACTTGTCCA-3‘	*M. quadriceps*	2.16	25
Insulin Receptor Substrate 1	*Irs1*	Mm.PT.58.43919344	5`-CCAGCATCAGCTTCCAGAA-3‘	*M. gastrocnemius*	2.06	26
			5‘-TGTGAATTGTGAAATAGTTCGAGTC-3‘	*M. quadriceps*	2.02	27
**Myokine genes**						
**Gene Name**	**Gene Abbreviation**	**PrimeTime qPCR Primer Assay**	**Sequence F/R**	**Tissue**	**PCR Efficiency**	**Quantification cycle value (Cq)**
Myostatin	*Mstn*	Mm.PT.58.13573446	5‘-GCCATGATCTTGCTGTAACCT-3‘	*M. gastrocnemius*	2.13	26
			5‘-CAGTCAAGCCCAAAGTCTCT-3‘	*M. quadriceps*	2.11	26
**Proteolysis genes**						
**Gene Name**	**Gene Abbreviation**	**PrimeTime qPCR Primer Assay**	**Sequence F/R**	**Tissue**	**PCR Efficiency**	**Quantification cycle value (Cq)**
F-Box Protein 32	*Fbxo32*	Mm.PT.58.7025875	5‘-TGAATAGCATCCAGATCAGCA-3‘	*M. gastrocnemius*	2.06	24
			5‘-GATGTTCAGTTGTAAGCACACAG-3‘	*M. quadriceps*	2.04	25
Tripartite Motif Containing 63	*Trim63*	Mm.PT.58.32840172	5‘-GCTACCTTCCTCTCAAGTGC-3‘	*M. gastrocnemius*	2.01	24
			5‘-CCTCTGCTATGTGTTCTAAGTCC-3‘	*M. quadriceps*	1.99	24
Ubiquitin B	*Ubb*	Mm.PT.58.29038744	5‘-CCAGTGGGCAGTGATGG-3‘	*M. gastrocnemius*	1.98	20
			5‘-GCTTACCATGCAACAAAACCT-3‘	*M. quadriceps*	2.01	20
**Protein synthesis genes**						
**Gene Name**	**Gene Abbreviation**	**PrimeTime qPCR Primer Assay**	**Sequence F/R**	**Tissue**	**PCR Efficiency**	**Quantification cycle value (Cq)**
AKT Serine/Threonine Kinase 1	*Akt1*	Mm.PT.58.8333433	5‘-GACGTAGCCATTGTGAAGGAG-3‘	*M.gastrocnemius*	2.20	28
			5‘-GCCGTTCCTTGTAGCCAAT-3‘	*M.quadriceps*	2.12	28
Phosphoinositide-3-Kinase Regulatory Subunit 1	*Pik3r1*	Mm.PT.58.5285167	5‘-AGAAAGGACTGGAATGTTCGAC-3‘	*M.gastrocnemius*	2.25	27
			5‘-GCATCTGCTAAGACGTGTAGC-3‘	*M.quadriceps*	2.17	26

Mitochondrial regulation genes						
**Gene Name**	**Gene Abbreviation**	**Reference**	**Sequence F/R**	**Tissue**	**PCR Efficiency**	**Quantification cycle value (Cq)**
Carnitine palmitoyltransferase 1B	*Cpt1b*	Sasaki et al. (2021)	5‘-ACCGTGAAGAGATCAAGCCGGT-3‘	*M.gastrocnemius*	1.88	27
			5‘-TCTCTTTGCCTGGGATGCGTGT-3‘	*M.quadriceps*	1.85	27
Peroxisome proliferator-activated receptor gamma coactivator 1-alpha	*Pgc-1α*	Sasaki et al. (2021)	5‘-TTCTGGGTGGATTGAAGTGGTG-3‘	*M.gastrocnemius*	1.91	26
			5‘-TGTCAGTGCATCAAATGAGGGC-3‘	*M.quadriceps*	1.90	27
**Reference genes**						
**Gene Name**	**Gene Abbreviation**	**PrimeTime qPCR Primer Assay**	**Sequence F/R**	**Tissue**	**PCR Efficiency**	**Quantification cycle value (Cq)**
Beta-2-Microglobulin	*B2m*	Mm.PT.39a.22214835	5‘-TGGTCTTTCTGGTGCTTGTC-3‘	*M.gastrocnemius*	2.09	24
			5‘-GGGTGGAACTGTGTTACGTAG-3‘	*M.quadriceps*	2.15	24
Peptidylprolyl Isomerase A	*Ppia*	Mm.PT.39a.2.gs	5‘-CAAACACAAACGGTTCCCAG-3‘	*M.gastrocnemius*	2.09	24
			5‘-TTCACCTTCCCAAAGACCAC-3‘	*M.quadriceps*	2.13	24
Ribosomal Protein Lateral Stalk Subunit P0	*Rplp0*	Mm.PT.58.43894205	5‘-TTATAACCCTGAAGTGCTCGAC-3‘	*M.gastrocnemius*	2.03	23
			5‘-CGCTTGTACCCATTGATGATG-3‘	*M.quadriceps*	2.01	22
RNA Polymerase II Subunit A	*Polr2a*	Mm.Pt.39a.22214849	5‘-GGTCCTTCGAATCCGCATC-3‘	*M.gastrocnemius*	2.15	28
			5‘-CAGGGTCATATCTGTCAGCATG-3‘	*M.quadriceps*	2.14	28

Reference:

Sasaki, Takashi, et al. "Muscle-specific TGR5 overexpression improves glucose clearance in glucose-intolerant mice." Journal of Biological Chemistry 296 (2021).

Fig. S1

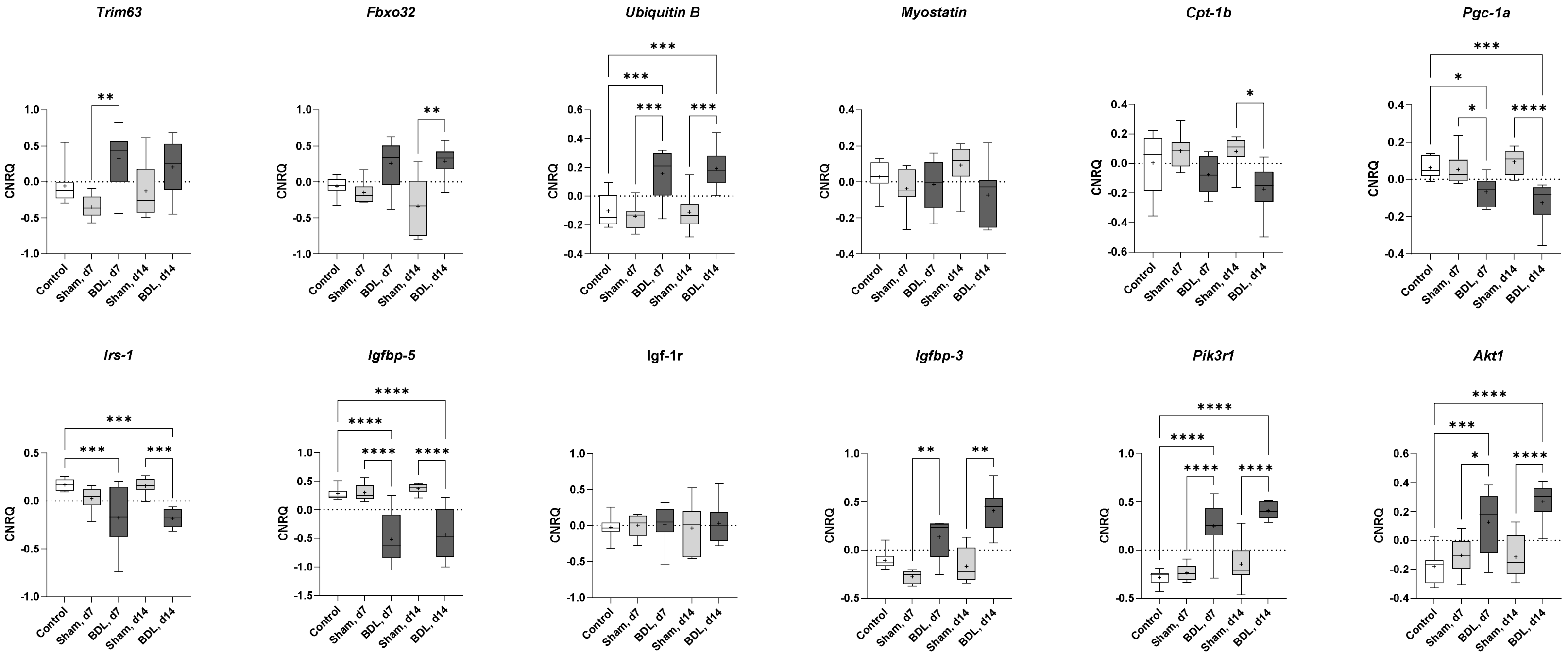

Figure S1 Effects of BDL on the expression of anabolic and catabolic genes in the gastrocnemius muscle. Mice underwent BDL or sham surgery and were sacrificed on postoperative days 7 (d7) and 14 (d14). Mice without surgery served as controls. Gene expression in the gastrocnemius muscle was quantified by real-time PCR as described in the Methods section. Relative gene expression levels are expressed as CNRQ (calibrated normalized relative quantity) values. Data are shown as box plots (min-max) with median (horizontal line) and mean (dot). n=8 mice per group; *p<0.05, **p<0.01, ***p<0.001, ****p<0.0001. One-way ANOVA with Šídák's multiple comparisons test (*Ubiquitin B*, *Myostatin*, *Pgc-1α, Insuline receptor substrate 1* (*Irs-1*), *Insulin-like growth factor binding protein 5* (*Igfbp-5*), *Akt1*, *Phosphatidylinositol 3-kinase r1 subunit* (*Pik3r1*)); Kruskal-Wallis-Test with Dunn's multiple comparisons test (all other genes).

Fig. S2

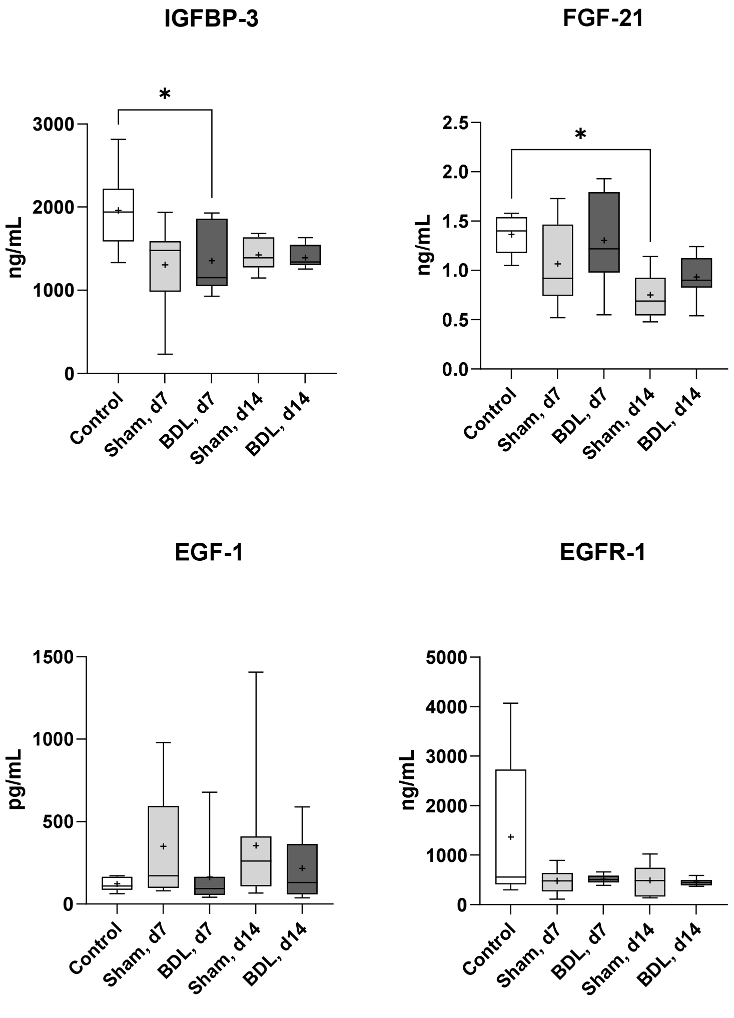

Figure S2 Plasma protein concentrations of mediators of inflammation, catabolic, and anabolic processes. Mice with BDL or sham surgery were sacrificed on postoperative days 7 (d7) and 14 (d14). Mice without surgery served as controls. The plasma protein levels were determined by Luminex assays and ELISA, respectively, as described in the Methods section. Data are shown as box plots (min-max) with median (horizontal line) and mean (dot). n≥7 mice per group (control n=5); *p<0.05. One-way ANOVA with Šídák's multiple comparisons test (FGF-21); Kruskal-Wallis-Test with Dunn's multiple comparisons test (all other proteins).
